# Myeloid DLL4 Does Not Contribute to the Pathogenesis of Non-Alcoholic Steatohepatitis in Ldlr^-/-^ Mice

**DOI:** 10.1371/journal.pone.0167199

**Published:** 2016-11-29

**Authors:** Mike L. J. Jeurissen, Sofie M. A. Walenbergh, Tom Houben, Tim Hendrikx, Jieyi Li, Yvonne Oligschlaeger, Patrick J. van Gorp, Marion J. J. Gijbels, Albert Bitorina, Isabell Nessel, Freddy Radtke, Marc Vooijs, Jan Theys, Ronit Shiri-Sverdlov

**Affiliations:** 1 Departments of Molecular Genetics, Pathology and Radiotherapy, School of Nutrition and Translational Research in Metabolism (NUTRIM), School for Cardiovascular Diseases (CARIM) and MAASTRO/School for Developmental Biology & Oncology (GROW), Maastricht University Medical Centre+, Maastricht, The Netherlands; 2 Experimental Vascular Biology, Department of Medical Biochemistry, Academic Medical Center, University of Amsterdam, Amsterdam, The Netherlands; 3 Ecole Polytechnique Fédérale de Lausanne, School of Life Sciences, Swiss Experimental Cancer Research Institute, Lausanne, Switzerland; Brigham and Women's Hospital, UNITED STATES

## Abstract

Non-alcoholic steatohepatitis (NASH) is characterized by liver steatosis and inflammation. Currently, the underlying mechanisms leading to hepatic inflammation are not fully understood and consequently, therapeutic options are poor. Non-alcoholic steatohepatitis (NASH) and atherosclerosis share the same etiology whereby macrophages play a key role in disease progression. Macrophage function can be modulated via activation of receptor-ligand binding of Notch signaling. Relevantly, global inhibition of Notch ligand Delta-Like Ligand-4 (DLL4) attenuates atherosclerosis by altering the macrophage-mediated inflammatory response. However, the specific contribution of macrophage DLL4 to hepatic inflammation is currently unknown. We hypothesized that myeloid DLL4 deficiency in low-density lipoprotein receptor knock-out (*Ldlr*^*-/-*^) mice reduces hepatic inflammation. Irradiated *Ldlr*^*-/-*^ mice were transplanted (tp) with bone marrow from wild type (Wt) or DLL4^f/f^LysMCre^+/0^ (DLL4^del^) mice and fed either chow or high fat, high cholesterol (HFC) diet for 11 weeks. Additionally, gene expression was assessed in bone marrow-derived macrophages (BMDM) of DLL4^f/f^LysMCre^WT^ and DLL4^f/f^LysMCre^+/0^ mice. In contrast to our hypothesis, inflammation was not decreased in HFC-fed DLL4^del^-transplanted mice. In line, *in vitro*, there was no difference in the expression of inflammatory genes between DLL4-deficient and wildtype bone marrow-derived macrophages. These results suggest that myeloid DLL4 deficiency does not contribute to hepatic inflammation *in vivo*. Since, macrophage-DLL4 expression in our model was not completely suppressed, it can’t be totally excluded that complete DLL4 deletion in macrophages might lead to different results. Nevertheless, the contribution of non-myeloid Kupffer cells to notch signaling with regard to the pathogenesis of steatohepatitis is unknown and as such it is possible that, DLL4 on Kupffer cells promote the pathogenesis of steatohepatitis.

## Introduction

NASH is characterized by an increase in fat accumulation (steatosis) and inflammation in the liver. The prevalence of steatosis is estimated to be ranging from 84% to 96% whereas in this population the prevalence of NASH is ranging from 25% to 55% [1]. Although steatosis is a rather benign and reversible condition, the presence of inflammation is the key feature of NASH, which can lead to further disease progression and eventually lead to liver cirrhosis [2, 3]. The exact mechanisms leading to hepatic inflammation are unknown and more insights are needed in order to discover novel therapeutic strategies.

Current research recognizes the critical role of Notch signaling in the context of immune cells [4]. Notch signaling occurs upon interaction of Notch receptors (e.g. Notch-1, -2, -3 or -4) on signal receiving cells and their membrane ligands (e.g. Jagged-1 (J1), Jagged-2 (J2), Delta-Like Ligand-1 (DLL1), Delta-Like Ligand-3 (DLL3) or Delta-Like Ligand-4 (DLL4)) on signal sending cells. Notch signaling has been implicated in the innate and adaptive immunity, which play an important role in various metabolic disorders [4–7]. An increasing amount of evidence point towards the existence of a shared inflammatory etiology between NASH and atherosclerosis with a central role for macrophages [8]. *Fukuda et al*. showed that global inhibition of DLL4 ameliorates atherosclerosis by altering macrophage-induced inflammatory responses, suggesting the importance of DLL4 on macrophage-mediated vascular inflammation [9, 10]. In NASH, Kupffer cells (KCs), the resident macrophages of the liver, play a central role in the initiation of hepatic inflammation and disease progression [8, 11, 12]. Relevantly, it was shown that Notch downstream targets are positively correlated with steatosis and inflammation in a cohort of non-alcoholic fatty liver disease (NAFLD) patients [13]. Furthermore, hepatic Notch activation lead to lipogenic gene expression and steatosis in chow fed mice whereas (DLL4)-Notch signaling promotes a fatty liver [9, 10, 14].

So far, the exact contribution DLL4-Notch in macrophages has not been investigated in the context of NASH. We hypothesized that myeloid DLL4 deficiency in low-density lipoprotein receptor knock-out (*Ldlr*^*-/-*^) mice reduces hepatic inflammation. To test this hypothesis, bone marrow of wild-type (Wt) or myeloid DLL4-deficient (DLL4^del^) mice was transplanted (-tp) into lethally irradiated *Ldlr*^*-/*-^ recipient mice and were fed chow or HFC for 11 weeks after a recovery period of 9 weeks. In contrast to our expectations, myeloid deletion of DLL4 did not reduce hepatic inflammation. These results suggest that myeloid DLL4 deficiency does not contribute to hepatic inflammation *in vivo*. Since, macrophage-DLL4 expression in our model was not completely suppressed, it can’t be totally excluded that complete DLL4 deletion in macrophages might lead to different results. Nevertheless, the contribution of non-myeloid Kupffer cells to notch signaling with regard to the pathogenesis of steatohepatitis is unknown and as such it is possible that, DLL4 on Kupffer cells promote the pathogenesis of steatohepatitis.

## Materials and Methods

### Mice, bone marrow transplantation and diet

All animals were housed under standard conditions and had access to food and water *ad libitum*. The animal experiments were approved by the committee for Animal Welfare of Maastricht University and were performed according to Dutch regulations. The DLL4^flox/flox^ mice were kindly donated by Prof. Freddy Radtke [15], and crossbred with LysMCre mice [16] to generate the myeloid DLL4 specific knock-out mice. *Ldlr*^*-/-*^ mice were obtained from in-house breeding. To generate the myeloid DLL4 deficient *Ldlr*^*-/-*^ mice, bone marrow transplantation was performed. *Ldlr*^*-/-*^ mice received one week before and four weeks after irradiation antibiotic water containing 100 mg/l neomycin *(Gibco*, *Breda*, *the Netherlands)* and 6*10^4^ U/l polymycin B Sulphate *(Gibco*, *Breda*, *the Netherlands)*. One day before and on the day of the transplantation *Ldlr*^*-/-*^ mice were lethally irradiated with 6 Gray of γ-radiation, thus receiving 12 Gray in total. Lethally irradiated *Ldlr*^*-/-*^ mice were then injected with 1*10^7^ bone marrow cells donated from DLL4^f/f^LysMCre^WT^ (Wt) or DLL4^f/f^LysMCre^+/0^ (DLL4^del^) mice. In order the fully ensure bone marrow replacement mice had a nine week recovery period. After nine weeks of recovery, transplanted (-tp) mice received either a chow (Wt-tp: n = 12, DLL4^del^-tp: n = 12) or HFC (Wt-tp: n = 20, DLL4^del^-tp: n = 20) diet, containing 21% butter and 0.2% cholesterol *(diet 1635; Scientific Animal Food and Engineering*, *Villemoissonsur-Orge*, *France)* for 11 weeks. Blood was collected form the tail vein at the end of the experiment and mice were sacrificed afterwards. Liver tissue was harvested and snap-frozen in liquid nitrogen or fixed in 4% formaldehyde/PBS.

### Bone marrow efficiency

In order to determine of the chimerism in the transplanted mice, we used donor bone marrow which has an *Ldlr*^WT^ origin, whereas recipient bone marrow an *Ldlr*^*-/-*^ origin. Genomic DNA was isolated using the PureLink^®^ Genomic DNA (*K182002; ThermoFisher Scientific*). A standard curve was generated by mixing DNA from *Ldlr*^*-/-*^ and *Ldlr*^*WT*^ bone marrow cells at different ratios. Chimerism was determined by quantifying the amount of *Ldlr*^*-/-*^ DNA in samples from 70 μL peripheral blood. To standardize for the amount of input DNA, the non-relevant p50 gene was quantified. Samples were assayed in duplicate on a 7900HT real-time PCR system by using 25 ng DNA, SensiMix^™^ Sybr & Fluorescein kit (*QT615-05*, *Bioline*), according to the manufacturer's instructions.

*Ldlr*^*-/-*^ specific primers are forward 5′-GCTGCAACTCATCCATATGCA-3′ and reverse 5′GGAGTTGTTGACCTCGACTCTAGAG-3. Forward and reverse p50-specific primers are 5′ACCTGGGAATACTTCATGTGACTAA-3′ and 5′ACACCAGAAGTCCAGGATTATAGC-3′, respectively. A standard curve was generated by plotting the mean threshold cycle (Ct) ΔCt (Ct p50—Ct *Ldlr*^-/-^) against the logarithm of the percentage *Ldlr*^*-/-*^ and calculation of a regression line. Chimerism was calculated from the percentage of *Ldlr*^*-/-*^ DNA in the blood samples (representing the remaining recipient bone marrow), determined by applying the mean ΔCt of the sample to the standard curve. Efficiency of the bone marrow transplantation in both groups was approximately 99% (data not shown).

### Plasma/Liver lipid measurements

Plasma cholesterol and triglycerides were measured via enzymatic colorimetric assay according to the manufacturer protocol (*Cholesterol Liquicolor CHOD_PAD; Human #10028*, *Instruchemie*, *Delfzijl) (Sigma Triglyceride (GPO Trinder) kit (Sigma Tr0100))*. Absorbance was measured with the BioRad Benchmark Plate Reader *(170-6750XTU; Bio-Rad*, *Hercules*, *CA)*. To measure liver cholesterol and triglycerides, liver homogenates were made. About 40–50 mg of frozen liver tissue was homogenized in 1 ml SET buffer (250 mM Sucrose, 2 mM EDTA, 10 mMTris) with 1 mm glass beads *(art*. *11079110)* on the max setting of the Biospec Mini Bead Beater-1. Afterwards, samples underwent two freeze-thaw cycles for complete cell destruction. To optimize cell destruction, samples were taken through a 25Gx5/8” needle several times and a final thaw cycle was added. Total protein content was measured via bicinchoninic acid (BCA) assay *(23225; Pierce*, *Rockford*, *IL)*. Liver cholesterol and triglycerides were measured via enzymatic colorimetric assay *(Cholesterol Liquicolor CHOD_PAD; Human #10028*, *Instruchemie*, *Delfzijl) (Triglyceride Liquicolor CHOD_PAD; Human #10724*, *Instruchemie*, *Delfzijl)*

### Liver histology

Livers of Wt-tp and DLL4^del^-tp mice were embedded in paraffin and sections of 4 μm thick were cut. H&E staining was performed according to the manufacturer’s protocol. Slides were scored for steatosis and liver cell injury (e.g. necrosis, inflammation, bile duct formation) by an experienced mouse pathologist (MJJG). For immunohistological stainings, frozen mouse liver tissue was cryo-embedded in Tissue-Tek^®^ *(Sakura Finetek Europe B*.*V*., *Alphen aan den Rijn*, *The Netherlands)* and sections of 7 μm thick were cut. For immunohistological stainings, cryosections of the liver were dried and fixated in dry acetone for 15 min. To block endogenous peroxidase activity, sections were incubated with 3% H_2_O_2_ solution for about 5 min. Tissue sections were also treated with Avidin/Biotin solution *(Vector; SP2001)* for 30 min. Afterwards, sections were incubated for 1 hour at room temperature (RT) with primary antibody for infiltrated macrophages (MAC-1; MAB1124; clone M1/70; 1:500) or Neutrophils (NIMP-1; rat anti-mouse Ly6-C, supernatant; clone: NIMP-R14, 1:100). Subsequently, tissue sections were incubated with secondary antibody *(Rabbit anti-Rat IgG Biotin (6180–08)*, *SouthernBiotech*, *Birmingham*, *AL*, *USA)* for 1 hour at RT. To amplify the signal, sections were incubated for 30 min in Peroxidase Vectastain Elite ABC solution (*Vector Laboratories*, *PK-6100*, *Peterborough*, *United Kingdom)*. For detection of the secondary antibody, the Peroxidase Substrate kit AEC (*Vector Laboratories*, *SK-4200*, *Peterborough*, *United Kingdom)* was used. Slides were counterstained with heamatoxylin. Pictures were taken with a Nikon digital camera DMX1200 and ACT-1 v2.63 software *(Nikon Instruments Europe*, *Amstelveen*, *The Netherlands)*. Immune cells were counted in 6 microscopical views (original magnification, 200x) and were noted as cells/square millimeter.

### Kupffer cell isolation

Whole liver (n = 4) of each experimental group were digested individually in digestion buffer (33.9 μg/ml Liberase TM, 0.002% DNaseI) for 20 min at 37°C. The digested liver solution was further disrupted by pushing it through a 100 μm cell strainer using wash buffer (PBS, 1% FCS and 2.5 mM EDTA). Cells were then centrifuged at 1500 rpm for 10 min at 4°C. Pellet was resuspended in wash buffer, removal of hepatocytes was accomplished by one low-spin centrifugation step at 300 rpm for 3 min. Supernatant, which was lysed from red blood cells, was collected and centrifuged. Next, Kupffer cells were isolated from the supernatant by using magnetic beads coated with a macrophage-specific monoclonal antibody (F4/80-APC, 1 μl/80x10^6^ cells) *(Biolegend)* and incubated for 20 min at 4°C. Afterwards, cells were washed and incubated with anti-APC microbeads (200 μl/100x10^6^ cells) *(Militenyi Biotec*, *Auburn*, *CA)* for 20 min at 4°C in the dark. After washing, samples were run into LS columns, put on a Quadro MACS magnet *(Militenyi Biotec*, *Auburn*, *CA)* and rinsed with wash buffer. Positively selected cells were flushed using wash buffer and collected for further analysis.

### RNA isolation and quantitative polymerase chain reaction

Total RNA was isolated from frozen mouse liver and Kupffer cells as described previously [17, 18]. To isolate total RNA from BMDM, FavorPrep^™^ Blood/Culture cell total RNA purification mini kit *(FABRK001*, *Favorgen*, *Vienna*, *Switzerland)* was used. First-strand complementary DNA (cDNA) was made from 500 ng total RNA of each mouse according to the manufacturer’s protocol *(iScript*^*™*^ *cDNA Synthesis Kit (170–8891)*, *Bio-Rad*, *Veenendaal*, *The Netherlands)*. As for total RNA of isolated Kupffer cells, approximately 50 ng of total RNA was used. Relative quantitative gene expressions of inflammatory markers were measured by quantitative PCR on an SDS 7900HT by using SensiMix SYBR HIROX (*Cat No QT605-05 Bioline*, *London*, *United Kingdom*) and 10 ng of cDNA template. For normalization, the geometric mean of two references genes were used (Cyclophillin A and ribosomal protein S12). Primers sets were developed with Primer Express version 2.0 *(Applied Biosystems)* using default settings. The primer sequences can be found in Table 1. Data from qPCR were analyzed with the LinRegPCR, Analysis of RT PCR data, Version 2015.3 [19–21].

**Table 1:** Primer sequences.

Gene	Primer forward	Primer reverse
**Cyclophillin A**	TTCCTCCTTTCACAGAATTATTCCA	CCGCCAGTGCCATTATGG
**S12**	GGAAGGCATAGCTGCTGGAGGTGT	CCTTCGATGACATCCTTGGCCTGA
**Abca1**	CCCAGAGCAAAAAGCGACTC	GGTCATCATCACTTTGGTCCTTG
**Abcg1**	TCGGACGCTGTGCGTTTT	CCCACAAATGTCGCAACCT
**Cd36**	GCCAAGCTATTGCGACATGA	AAAAGAATCTCAATGTCCGAGACTTT
**LXRα**	CAACAGTGTAACAGGCGCT	TGCAATGGGCCAAGGC
**Notch-1**	AGGACCTCATCAACTCACACGC	TCTTTGTTAGCCCCGTTCTTCAG
**Notch-2**	CCGTGTTGACTTCTGCTCTCTCAC	CCTACTACCCTTGGCATCCTTTG
**Notch-3**	TCTCAGACTGGTCCGAATCCAC	ACACTTGCCTCTTGGGGGTAAC
**Notch-4**	ATGCGAGGAAGATACGGAGTGG	TCGGAATGTTGGAGGCAGAAC
**Dll1**	CTACTACGGAGAGGGCTGCT	CCAGGGTTGCACACTTTCTC
**Dll4**	ACAACTTGTCGGACTTCCAG	CAGCTCCTTCTTCTGGTTTG
**Jagged-1**	ATCGTGCTGCCTTTCAGTTT	ACTGTCAGGTTGAACGGTGTC
**Jagged-2**	GTCGTCATCCCCTTCCAGT	CTCCTCATTCGGGGTGGTAT
**Hey1**	GAAACTTGAGTTCGGCTCTAGG	GCTTAGCAGATCCTTGCTCCAT
**Hes1**	AGGCGGACATTCTGGAAATG	CGGTACTTCCCCAGCACACTT
**Tnf-α**	CATCTTCTCAAAATTCGAGTGACAA	TGGGAGTAGACAAGGTACAACCC
**Itgam**	ACTTTCAGAAGATGAAGGAGTTTGTCT	TGTGATCTTGGGCTAGGGTTTC
**Icam**	CTACCATCACCGTGTATTCGTTTC	CGGTGCTCCACCATCCA

### Cell culture

Bone marrow was isolated from hind limbs of DLL4^f/f^LysMCre^WT^ (Wt; n = 3) and DLL4^f/f^LysMCre^+/0^ (DLL4^del^; n = 3) mice. In short, the femur and tibiae were flushed with cold PBS. Bone marrow cells were cultured for 8 days in RPMI1640 cell culture medium (10% Fetal Calf Serum (FCS) *(Bodinco BV*, *Alkmaar*, *The Netherlands)*, 1% penicillin/streptomycin, 1% L-Glutamine, 20mM HEPES) *(GIBCO by Life technologies*, *Bleiswijk*, *the Netherlands)* supplemented with 20% LCM (L929 cell conditioned medium which contains M-CSF) to differentiate into BMDM. All cells were cultured at 37°C in the presence of 5% CO_2_ atmosphere. Cells were seeded in a 24-wells plate *(Greiner*, *662102*, *Alphen a/d Rijn)* (350,000 cells/well) and stimulated for 4 hours with LPS (100 ng/ml). For experiments with immobilized DLL4, cells were coated overnight with recombinant DLL4 (1 μg/ml; *R&D Systems*) and 0.2% gelatin in PBS at 4°C. Wells were rinsed once with PBS before plating Wt BMDM (350.000 cells/well) followed by 4 hrs incubation at 37°C. Tumor necrosis factor alpha (TNFα) protein was measured via ELISA *(88-7324-88; Affymetrix*, *eBioscience*, *Vienna*, *Austria*) according to the manufacturer’s protocol.

### Western blot

BMDM of Wt and DLL4^del^ mice were lysed in RIPA buffer (50 mM Tris-HCL pH 7.5, 150 mM NaCl, 0.5% Sodium deoxycholate, 1% Triton X-100, 0.1% SDS) supplemented with protease and phosphatase inhibitor mixture. For making liver homogenates, about 40–50 mg of frozen liver tissue was homogenized in 1 ml RIPA About 40–50 mg of frozen liver tissue was homogenized in 1 ml SET buffer (250 mM Sucrose, 2 mM EDTA, 10 mMTris) with 1 mm glass beads *(art*. *11079110)* on the max setting of the Biospec Mini Bead Beater-1. To optimize cell destruction, samples were taken through a 25Gx5/8” needle several times and a final thaw cycle was added. Total protein content was measured via bicinchoninic acid (BCA) assay *(23225; Pierce*, *Rockford*, *IL)*. For western blot analysis, equal amounts of protein (30 μg) were loaded on the gel. After SDS/PAGE electrophoresis, protein was transferred on nitrocellulose membrane (*Biorad*). The membrane was blockade with 5% non-fat dry milk for 1 hr at room temperature. Afterwards, the membrane was incubated overnight at 4°C with primary antibody against DLL4 *(0*.*3 ug/ml*, *ab183532*, *Cambridge*, *United Kingdom)* or β-actin *(1*:*1000 dilution*, *Cell Signaling Technology*, *Danvers*, *MA*, *USA)* which was us as a reference protein. Detection was performed according to its primary antibody using anti-goat (*Santa Cruz*) or anti-rabbit (*Cell Signaling*) horse-radish peroxidase (HRP)-conjugated secondary antibodies, followed by chemiluminescence.

### Statistical analysis

Significant differences between the experimental groups were analyzed with the two-way ANOVA followed by a Tukey post-hoc test using the IBM^®^ SPSS Statistics program (Version 22.0.0.). *In vitro* results were analyzed for significant differences with the two-tailed unpaired t-test using GraphPad Prism (Version 5.03). Outliers were determined via the Grubbs’ Test. Data were expressed as the mean ±SEM and considered significant at p < 0.05. *, ** and *** indicate p < 0.05, 0.01 and 0.001 resp.

## Results

### Myeloid DLL4 deficiency has no effect on plasma and liver lipid levels

**Fig 1:**
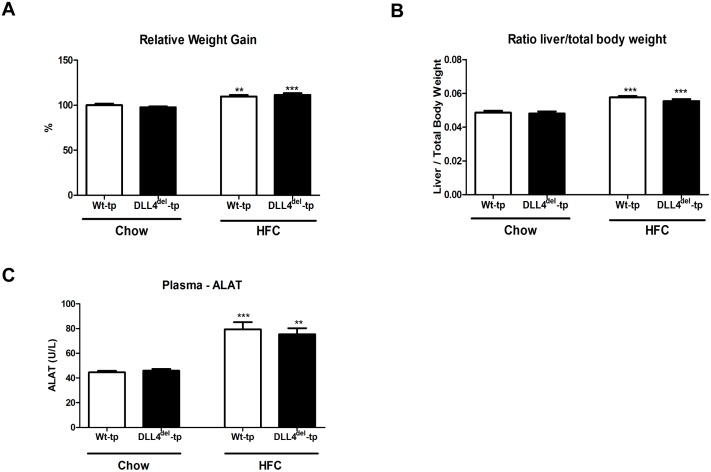
Body and liver weights of Wt-tp and DLL4^del^-tp mice. **(A)** Relative weight gain was calculated from the body weights of Wt-tp and DLL4^del^-tp mice. (**B)** Relative liver/total body ratio was measured from the liver and body weights of Wt-tp and DLL4^del^-tp mice. (**C)** Plasma ALT levels were measured of Wt-tp and DLL4^del^-tp mice. All data are represented as mean +/- SEM. Data are significant at * p< 0.05, ** p< 0.01, *** p< 0.001. Significance is compared to the chow group of the respective genotype.

**Fig 2:**
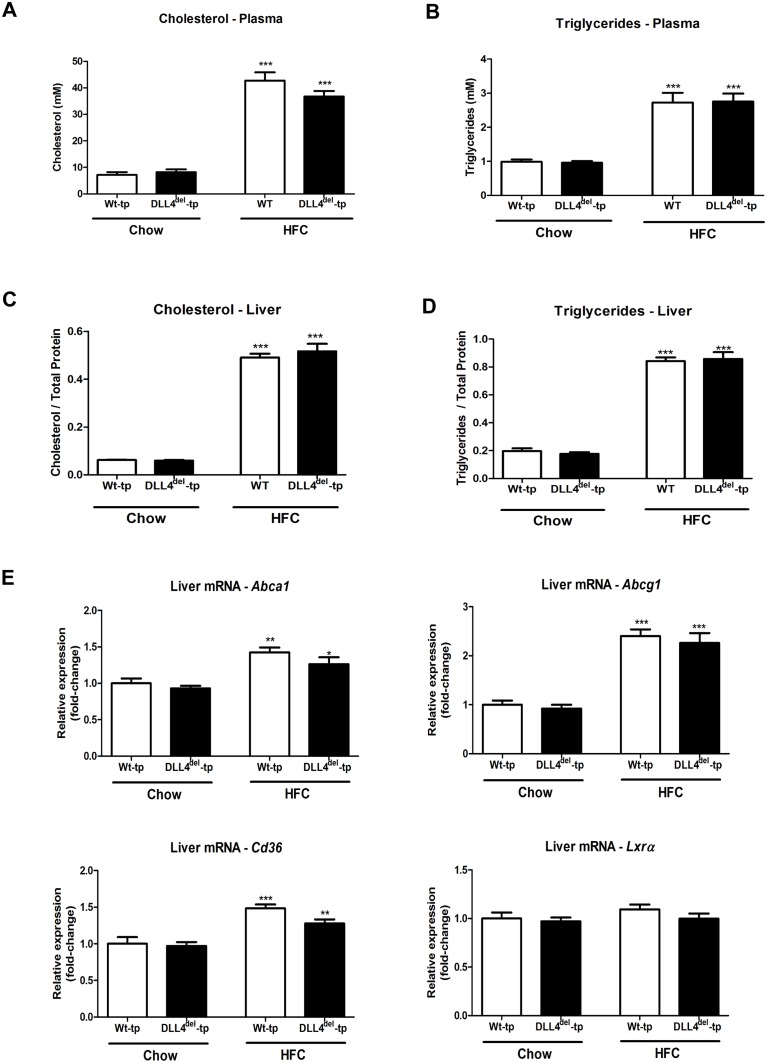
Lipid level measurements in Wt-tp and DLL4^del^-tp mice. **(A-D)** Cholesterol and triglyceride levels were measured in plasma and in the liver of Wt-tp and DLL4^del^-tp mice. (**E)**. Gene expression of *Abca1*, *Abcg1*, *Cd36 and Lxrα* were measured in the liver of Wt-tp and DLL4^del^-tp mice. All data are represented as mean +/- SEM. Data are significant at * p< 0.05, ** p< 0.01, *** p< 0.001. Significance is compared to the chow group of the respective genotype.

To investigate the effect of myeloid DLL4 deficiency on the health status of Wt-tp and DLL4^del^-tp mice, relative weight gain and liver/body weight ratio were determined. As expected, upon HFC, both the relative weight gain and liver/body weight ratio were increased compared to chow. No differences were observed between Wt-tp and DLL4^del^-tp mice (Fig 1A and 1B). To determine the extent of liver damage, the liver enzyme alanine aminotransferase (ALT) was measured. Upon HFC feeding, ALT levels were increased in Wt-tp and DLL4^del^-tp mice compared to chow, but levels remained similar between both groups (Fig 1C). Next, we investigated the effect of myeloid DLL4 on plasma and liver lipid levels. Upon HFC feeding, cholesterol and triglyceride levels were increased in Wt-tp and DLL4^del^-tp mice. However, no significant differences were observed between both groups in both plasma and liver (Fig 2A–2D). To further determine the effects of myeloid DLL4 deficiency on cholesterol metabolism, gene expression of ATP-binding cassette subfamily A member 1 (*Abca1*), ATP-binding cassette subfamily G member 1 (*Abcg1*), Liver X receptor alpha (*Lxrα*) and Cluster of Differentiation 36 (*Cd36*) were analyzed in the livers of Wt-tp and DLL4^del^-tp mice. Gene expression of *Abca1* and *Cd36* were significantly upregulated in the livers of Wt-tp and DLL4^del^-tp mice on an HFC diet compared to chow-fed mice. Similar hepatic mRNA levels were detected between Wt-tp and DLL4^del^-tp mice when fed chow or HFC (Fig 2E). Altogether, these data suggest that myeloid DLL4 signaling has no effect on lipid metabolism.

### Hepatic inflammation is not changed in myeloid DLL4-deficient mice

**Fig 3:**
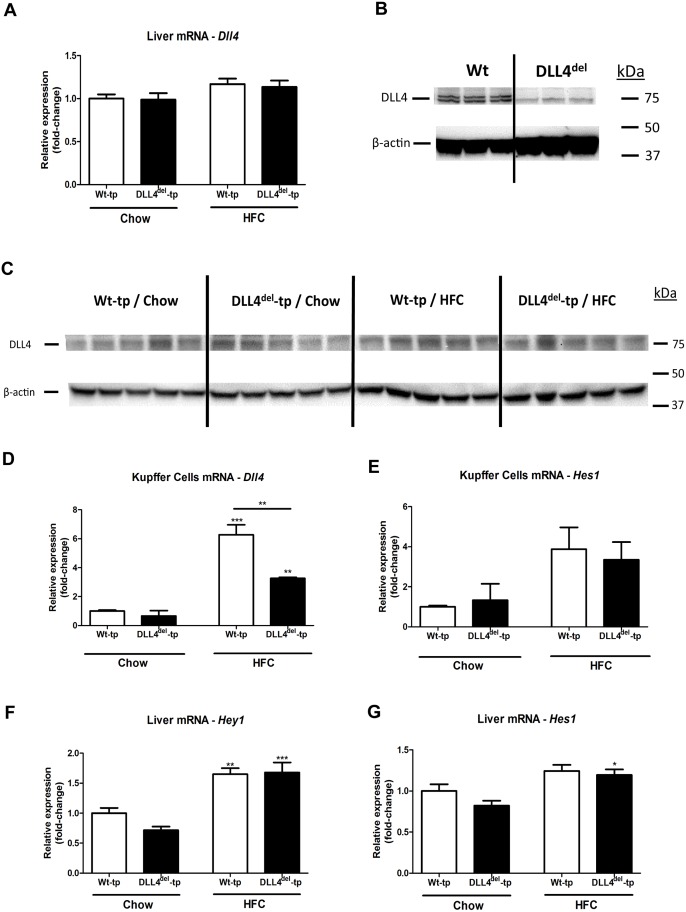
Hepatic gene and protein expression analysis of DLL4 and its downstream targets. **(A)** Gene expression of *Dll4* in the livers of Wt-tp and DLL4^del^-tp mice. (**B-C)** DLL4 protein expression in BMDM and livers of mice, respectively. (**D-E)** Gene expression analysis of *Dll4* and *Hes1* in isolated KCs from Wt-tp and DLL4^del^-tp mice. (**F-G)** Gene expression of *Hey1* and *Hes1* in the livers of Wt-tp and DLL4^del^-tp mice. All data are represented as mean +/- SEM. Data are significant at * p< 0.05, ** p< 0.01, *** p< 0.001. Significance is compared to the chow group of the respective genotype.

**Fig 4:**
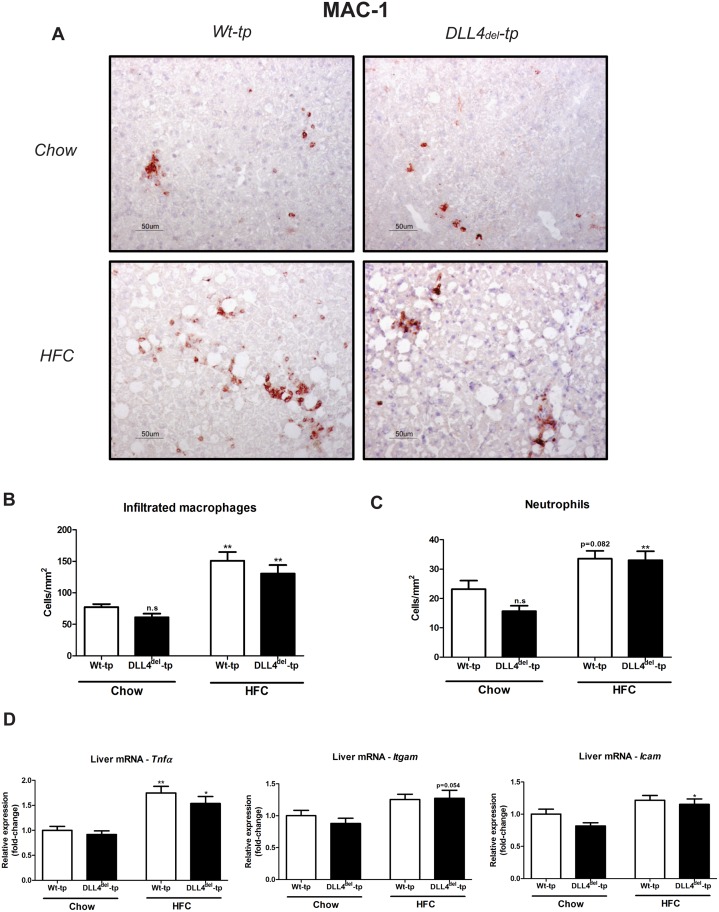
Hepatic inflammation in Wt-tp and DLL4^del^-tp mice. **(A)** Representative pictures of the MAC-1 staining on the livers of Wt-tp and DLL4^del^-tp mice. Original magnification: 200x. (**B-C)** Quantification of the MAC-1 and NIMP staining for infiltrated macrophages and amount of neutrophils, respectively. (**D)** Gene expression of *Tnfα*, *Itgam and Icam* were measured in the livers of Wt-tp and DLL4^del^-tp mice. All data are represented as mean +/- SEM. Data are significant at * p< 0.05, ** p< 0.01, *** p< 0.001. Significance is compared to the chow group of the respective genotype.

To investigate that DLL4 is knocked down specifically in myeloid cells, we first determined DLL4 expression in whole livers of Wt- and DLL4^del^–tp mice. We found that *Dll4* expression both on mRNA and protein level in whole livers was similar between Wt-tp and DLL4^del^-tp mice (Fig 3A and 3C, respectively). Next, protein expression of DLL4 was assessed in Wt and DLL4^del^ BMDM. In line with our gene expression data regarding DLL4 in Kupffer cells (Fig 3D), DLL4 protein expression was reduced in DLL4^del^ BMDM compared to Wt BMDM (Fig 3B). Altogether, these data indicate that DLL4 deficiency is selective for myeloid cells. To determine the effect on myeloid DLL4 deficiency on Notch signaling, the expression of Notch target genes was investigated in the livers of Wt-tp and DLL4^del^-tp mice. Gene expression analysis of the downstream targets of DLL4, Hairy/enhance of split-1 (*Hes1*) and Hairy/enhancer-of-split related with YRPW motif protein 1 (*Hey1*), was analyzed. Upon HFC feeding, *Hey1* and *Hes1* expression was increased in Wt-tp and DLL4^del^-tp mice, indicative for increased Notch signaling activation. However, no changes in *Hey1* and *Hes1* were observed between both groups (Fig 3F and 3G). Additionally, *Hes1* gene expression in KCs of Wt-tp and DLL4^del^-tp mice on chow and HFC diet was determined. No differences were observed in *Hes1* gene expression between KCs of Wt and DLL4^del^-tp mice (Fig 3E). Next, gene expression of Notch-receptors and ligands were measured in the livers of Wt-tp and DLL4^del^-tp mice. Upon HFC, gene expression of *Notch-1*, *Notch-3*, and *Jagged-1* was increased compared to chow-fed mice in both Wt-tp and DLL4^del^-tp mice, whereas *Dll1* gene expression was reduced. However, no differences were observed between Wt-tp and DLL4^del^-tp mice in either the chow or HFC group (S1 Fig). Similar findings were observed in BMDM of Wt and DLL4^del^ mice; upon LPS stimulation, the expression of *Notch-1*, *Notch-2* and *Dll1* was increased in both Wt and DLL4^del^ BMDM, whereas *Jagged-2* gene expression was reduced compared to non-stimulated conditions. No differences in Notch ligands and receptors were observed between Wt and DLL4^del^ BMDM (S2 Fig). Altogether, these findings suggest that hematopoietic deletion of DLL4 is not associated with changes in the expression of other Notch receptors or ligands. Next, to investigate whether myeloid DLL4 deficiency lowers hepatic inflammation, immunohistological stainings and gene expression analysis were performed on liver tissue of Wt-tp and DLL4^del^-tp mice. First, we performed an H&E staining on liver sections of Wt-tp and DLL4^del^-tp mice. These sections were scored for steatosis and liver cell injury (e.g. necrosis, inflammation, bile duct formation) by an experienced mouse pathologist in a blinded manner. Histological analysis revealed that, upon an HFC diet, steatosis and liver cell injury was pronounced in both Wt-tp and DLL4^del^-tp mice. These findings were also supported by ALAT plasma levels, which is a marker for liver injury (Fig 1C). In line with our previously obtained results, no differences were observed between the two genotypes (S3 Fig). These data further support the conclusion that myeloid DLL4 deficiency does not affect liver steatosis and hepatic inflammation. Additionally, liver sections were stained for infiltrated macrophages (MAC-1 staining) and neutrophils (NIMP staining). Upon HFC feeding, infiltrated macrophages were increased in Wt-tp and DLL4^del^-tp mice compared to chow-fed mice. Similar effects were observed for neutrophils. However, no differences were observed between the two genotypes in the chow- and HFC-fed group for infiltrated macrophages and neutrophils (Fig 4A–4C). Moreover, gene expression levels of *Tnfα*, Integrin Alpha M (*Itgam)* and Intercellular Adhesion Molecule 1 (*Icam)* were similar between Wt-tp and DLL4^del^-tp mice (Fig 4D). To determine foam cell formation a CD68 staining was performed on livers of Wt-tp and DLL4^del^-tp mice. There were no differences in foam cell formation between Wt-tp and DLL4^del^-tp HFC fed mice (data not shown). Overall, these results suggest that myeloid DLL4 deficiency does not lower hepatic inflammation.

### DLL4 deficiency does not affect inflammatory gene expression in bone marrow-derived macrophages

**Fig 5:**
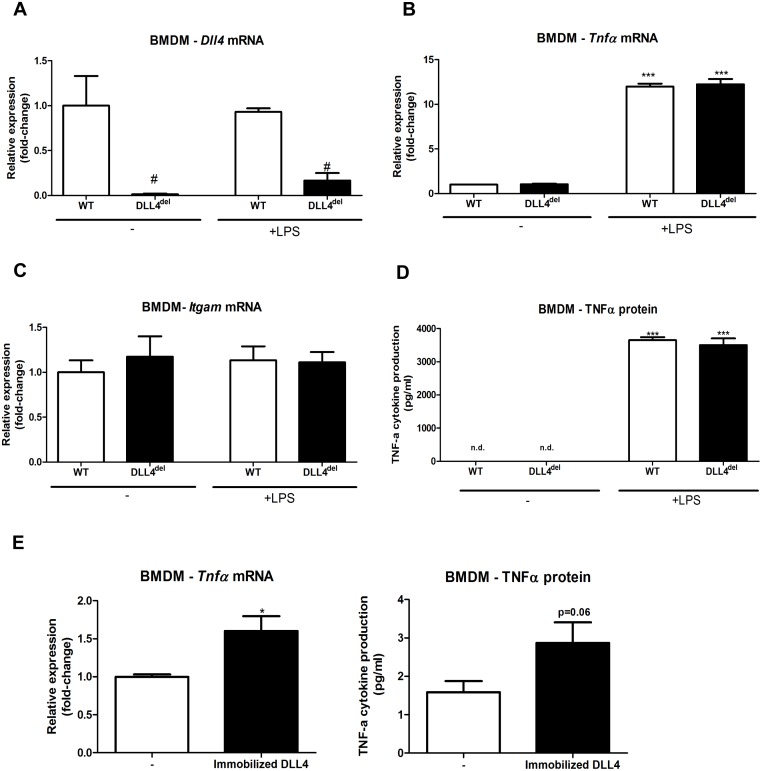
Inflammatory response of bone marrow-derived macrophages from Wt and DLL4^del^ mice. **(A)** Gene expression of *Dll4* was measured in BMDM of Wt and DLL4^del^ mice. (**B-C)** Gene expression of *Tnfα* and *Itgam* were measured in BMDM of Wt and DLL4^del^ mice. (**D)** TNFα cytokine production was measured in BMDM of Wt and DLL4^del^ mice. (**E)** Wt BMDM stimulated with immobilized recombinant DLL4. All data are represented as mean +/- SEM. Data are significant at * p< 0.05, ** p< 0.01, *** p< 0.001. Significance is compared to Wt BMDM.

As we did not observe differences on hepatic inflammation *in vivo*, we next investigated whether bone marrow-derived macrophages (BMDM) of myeloid DLL4-deficient mice are less susceptible for inflammation. Bone marrow from DLL4^f/f^LysMCre^Wt^ (Wt) and DLL4^f/f^LysMCre^+/0^ (DLL4^del^) was isolated and differentiated to BMDM followed by an LPS stimulus. As expected, DLL4^del^ BMDM showed a significant reduction of DLL4 expression when compared to Wt macrophages (Fig 5A). However, no significant differences in TNFα cytokine production, *Tnfα* and *Itgam* expression were detected when compared to Wt BMDM upon LPS stimulation (Fig 5B–5D). While the differences between the groups for *Dll4* and *Itgam* gene expression remain similar in the condition without LPS, TNFα cytokine production was not detectable. *Tnfα* gene expression levels were increased significantly in Wt and DLL4^del^ BMDM due to the LPS stimulus. These data show that DLL4^del^ BMDM can contribute to inflammation to the same extent as compared to Wt BMDM. To investigate the relative contribution of DLL4 to LPS-induced inflammation, DLL4 was immobilized in culture plates, where it acts as an inflammatory stimulus on Wt BMDM in the absence of LPS. Upon DLL4 stimulation, gene expression of *Tnfα* was significantly increased. A similar trend was observed in TNFα cytokine production. However, in the absence of LPS, the levels of TNFα cytokine production are extremely low (±1.5–3.0 pg/ml) (Fig 5E). These data suggest a minor role for myeloid DLL4 in triggering inflammation.

## Discussion

Notch signaling is involved in various metabolic diseases [7, 9, 13, 22] and has been described as an essential modulator for inflammation and macrophage function [7, 23–27]. While many Notch ligands have been investigated thoroughly, in the current study, we investigated for the first time the contribution of myeloid DLL4 in the context of hepatic inflammation.

Macrophage activation is essential for atherosclerotic plaque development [28–31] and recent studies have implicated Notch signaling in this process [9, 23, 32]. These studies showed that DLL4 ligand-expressing macrophages are found in human atherosclerotic lesions and that pro-inflammatory stimuli can increase DLL4 expression in macrophages [23]. Furthermore, it has been shown *in vivo* that plaque progression was reduced in apolipoprotein E-deficient mice after treatment with a γ-secretase, in order to inhibit Notch signaling [32]. Taken into account the prominent role of Notch signaling in atherosclerosis, and since NASH and atherosclerosis share similar disease mechanisms [8], the results obtained in the current study were unexpected. In contrast to our results, global inhibition of DLL4 using specific antibodies resulted in a reduction in plaque development and decreased fat accumulation in *Ldlr*^*-/-*^ mice. Next to that, they showed that F4/80 gene expression in the liver was reduced in these mice [9, 10], while our result showed no differences in hepatic inflammation. *In vitro* data from our group and others demonstrated the pro-inflammatory role of DLL4 on macrophages. However, data regarding the role of DLL4 on cells other than macrophages is lacking. In our experimental design, the DLL4 deletion is restricted to myeloid cells. In contrast, *Fukuda et al*. used a global inhibition of DLL4 (via anti-DLL4 antibodies) [9]. It is therefore possible that *in vivo*, the contribution of DLL4 on myeloid cells is minor and cells other than macrophages contribute to the inflammatory response, as observed by *Hoebe et al*. [33]. For example, when stromal cells, that express DLL4, were co-cultured with macrophages, the inflammatory response was increased, compared to stromal cells without DLL4 co-incubated with macrophages [23]. These data demonstrate the contribution of other DLL4-expressing cells to inflammation. In line, there is evidence that Notch receptors can be activated through other Notch ligands [34–37]. Furthermore, Notch-1, -2 and -3 are highly expressed on monocytes and macrophages and *in vitro* studies have shown that these cells can undergo cytokine specific apoptosis by interaction of DLL1 which could influence the macrophage inflammatory response [23, 25, 38]. Next to that, it can be speculated that DLL4-expressing hepatocytes may also affect myeloid DLL4-Notch signaling, as myeloid DLL4 signaling is mediated through all four Notch receptors [23, 39, 40]. Additionally, DLL4 induces Notch-1, -2, -3 cleavages [41]. As such it is likely that, macrophages in DLL4^del^-tp mice could still be activated via hepatic DLL4. Interestingly, *Koga et al*, showed that *Ldlr*^*-/-*^ hyperlipidemic mice showed high levels of soluble DLL4 in the plasma compared to Wt mice [42]. *Fung et al*, already showed that soluble DLL4 is able to activate Notch signaling in macrophages [23]. Based on these observations it can be suggested that in our model hepatocytes could still be functioning as suitable donor for DLL4 activation as they still express DLL4.

In conclusion, our data suggest that the inhibition of one single Notch-ligand in the myeloid linage is not sufficient to overcome hepatic inflammation. Nevertheless, since the macrophage-Dll4 expression in our model was not completely suppressed, it can’t be totally excluded that complete DLL4 deletion in macrophages might lead to different results. Furthermore, there is a possibility that Kupffer cell isolation using magnetic beads may contain other cells such as endothelial cells, which could explain for these findings. Finally, the contribution of non-myeloid Kupffer cells to notch signaling with regard to the pathogenesis of steatohepatitis is unknown and as such it is possible that, DLL4 on Kupffer cells promote the pathogenesis of steatohepatitis. Therefore, further research should emphasize on the effects of complete DLL4 deletion in myeloid cells and the contribution of non-myeloid cells to DLL4-Notch signaling.

## Supporting Information

S1 Fig Gene expression of Notch receptors/ligands in the livers of Wt-tp and DLL4^del^-tp mice. (TIF)

S2 Fig Gene expression of Notch receptors/ligands in BMDM from Wt and DLL4^del^ mice. (TIF)

S3 Fig Representative pictures of H&E staining on the livers of Wt-tp and DLL4^del^-tp mice. (TIF)

## Abbreviations

NASH: Non-alcoholic steatohepatitis
DLL: Delta-Like Ligand
Ldlr^-/-^: low-density lipoprotein receptor knock-out
tp: transplanted
Wt: Wild type
DLL4^del^: DLL4^f/f^LysMCre^+/0^
HFC: High fat diet
BMDM: Bone marrow-derived macrophages
J1: Jagged-1
KC: Kupffer cell
NAFLD: Non-alcoholic fatty liver disease
PBS: phosphate-buffered saline
BCA: bicinchoninic acid
FCS: Fetal calf serum
LCM: L929 conditioned medium
TNFα: Tumor necrosis factor alpha
ALT: Alanine aminotransferase
ABCa1: ATP-binding cassette subfamily A member 1
ABCg1: ATP-binding cassette subfamily G member 1
LXRα: Liver X receptor Alpha
CD36: Cluster of Differentiation 36
HEY1: Hairy/Enhancer of split related with YRPW motif protein 1
HES1: Hairy/Enhancer of split 1
Itgam: Integrin alpha 1
ICAM: Intracellular adhesion molecule-1
