# Supplementary figures and images for: Myeloid DLL4 Does Not Contribute to the Pathogenesis of Non-Alcoholic Steatohepatitis in Ldlr-/- Mice
Source: PLoS One. 2016 Nov 29;11(11):e0167199. doi: 10.1371/journal.pone.0167199 (PMC5127569; doi:10.1371/journal.pone.0167199)

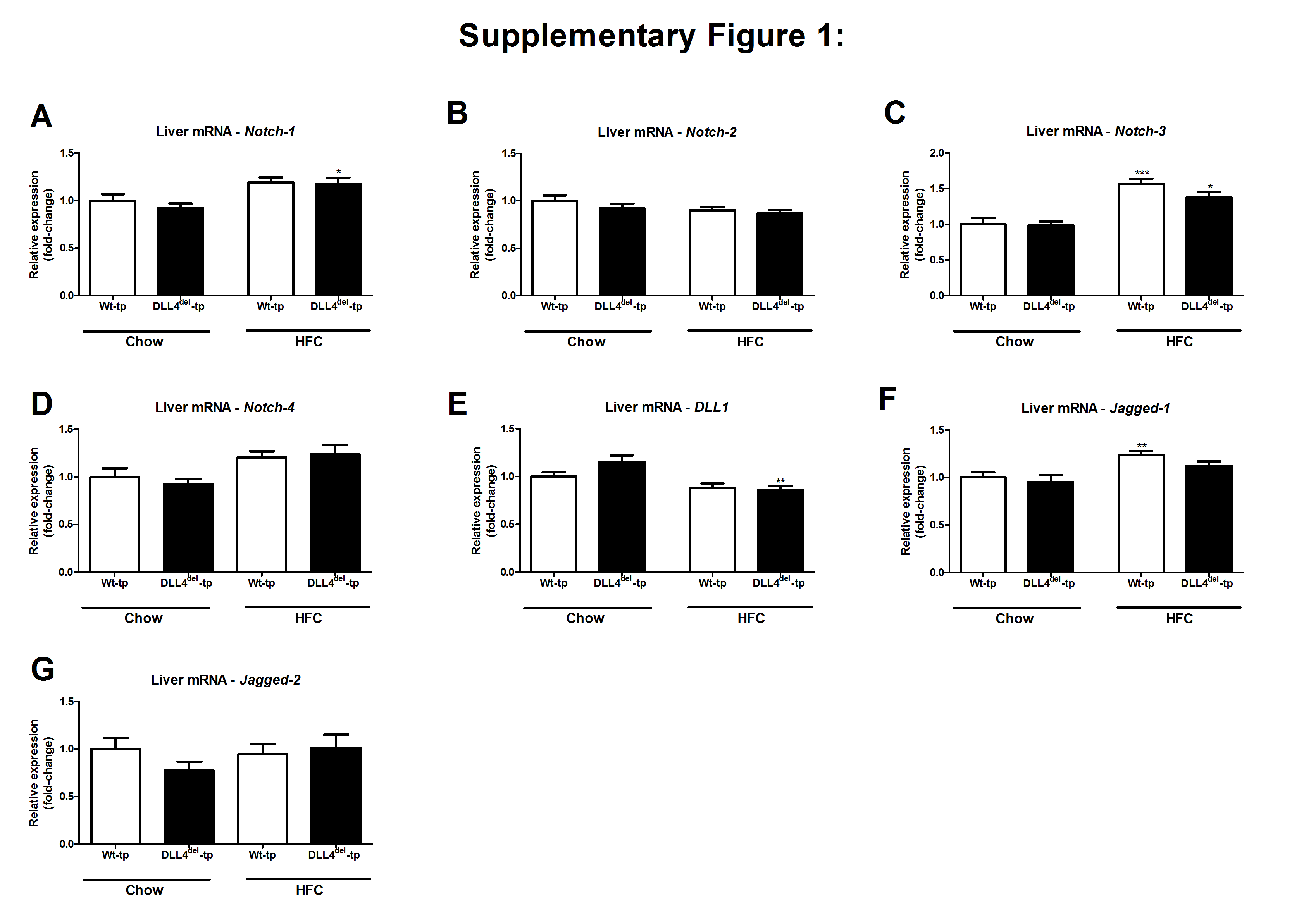

Supplement: S1 Fig — (TIF) [file pone.0167199.s001.tif]

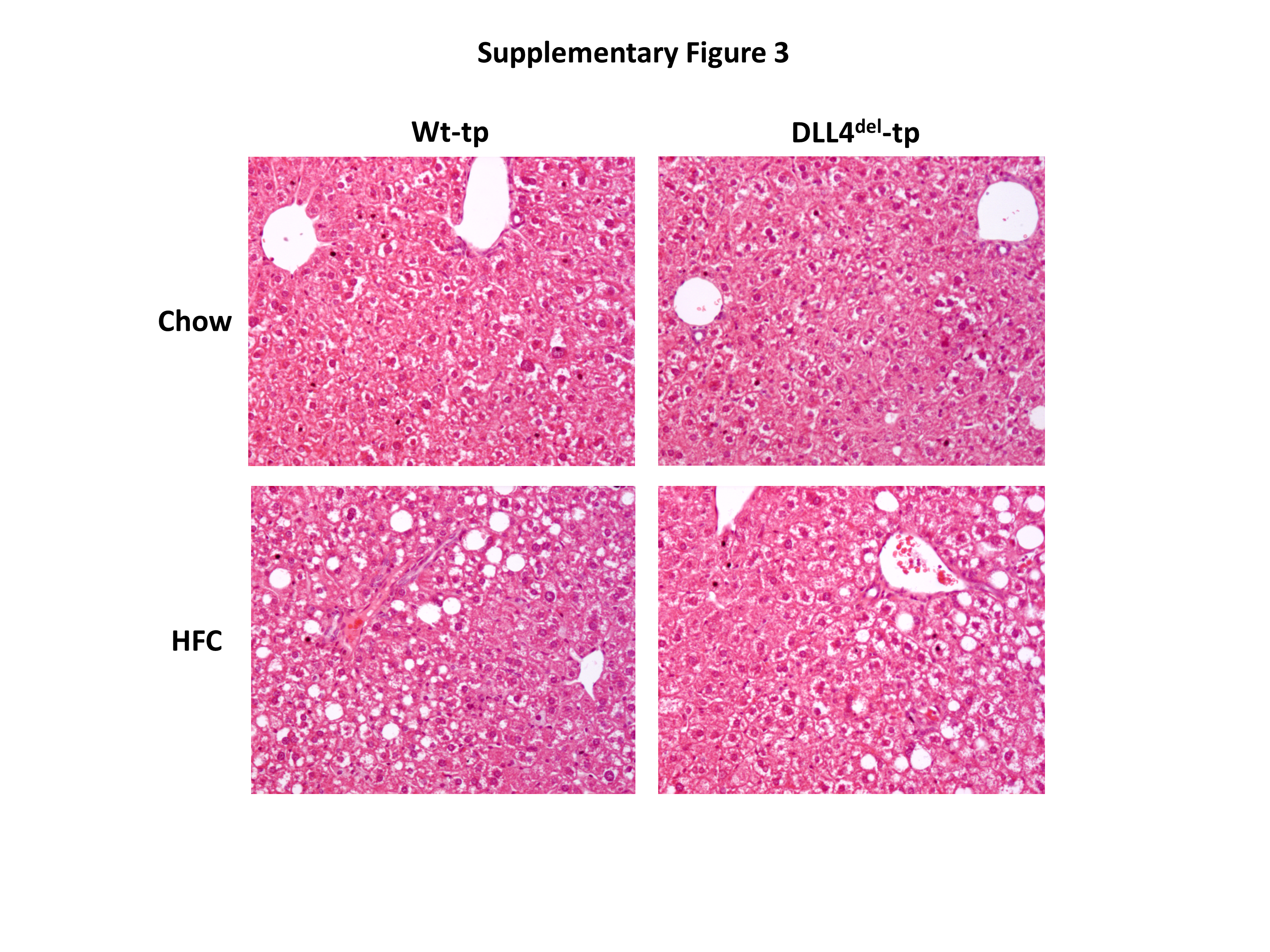

Supplement: S3 Fig — (TIF) [file pone.0167199.s003.tif]
